# Omega-3 Polyunsaturated Fatty Acids in Critical Illness: Anti-Inflammatory, Proresolving, or Both?

**DOI:** 10.1155/2017/5987082

**Published:** 2017-06-14

**Authors:** Alessio Molfino, Maria Ida Amabile, Massimo Monti, Maurizio Muscaritoli

**Affiliations:** ^1^Department of Clinical Medicine, Sapienza University of Rome, Viale dell'Università 37, 00185 Rome, Italy; ^2^Department of Surgical Sciences, Sapienza University of Rome, Viale Regina Elena 324, 00161 Rome, Italy

## Abstract

Prognosis and outcomes of critically ill patients are strictly related with inflammatory status. Inflammation involves a multitude of interactions between different cell types and chemical mediators. Omega-3 polyunsaturated fatty acids (PUFAs), mainly represented by eicosapentaenoic acid (EPA) and docosahexaenoic acid (DHA), are able to inhibit different pathways including leukocyte chemotaxis, adhesion molecule expression and interactions, and production of inflammatory cytokines, through the action of specialized proresolving mediators (SPMs). SPMs from omega-6 fatty acids, such as lipoxins, and from omega-3 fatty acids such as resolvins, protectins, and maresins, act in reducing/resolving the inflammatory process in critical diseases, stimulating the phases of resolution of inflammation. In this light, the resolution of inflammation is nowadays considered as an active process, instead of a passive process. In critical illness, SPMs regulate the excessive posttrauma inflammatory response, protecting organs from damage. This review focuses on the role of omega-3 PUFAs as pharma nutrition agents in acute inflammatory conditions, highlighting their effects as anti-inflammatory or proresolving agents.

## 1. Introduction

Polyunsaturated fatty acids (PUFAs) of the omega-3 series are essential nutrients since they cannot be produced by humans [1] and whose dietary intake, with food and/or supplements, is associated with several health benefits [2, 3]. Omega-3 PUFAs, primarily found in dietary fish oils [4], are derived also from plants [5] and are substrates able to reduce or limit inflammation in critical illness [6]. The underlying molecular mechanisms responsible for omega-3 PUFAs' biological effects are mediated by the production of proresolving mediators, which have been proposed to modulate and likely resolve inflammatory responses [7]. Lipid mediators synthesized from omega-6 and omega-3 fatty acids are known not only as anti-inflammatory molecules but also to have a key role in inducing active resolution of inflammation. These molecules are defined as specialized proresolving mediators (SPMs) [8]. In this light, PUFAs may be considered as potent modulators of the mechanisms regulating the onset, prolongation, and resolution of inflammation and, therefore, considered to be protective against uncontrolled inflammatory response. The omega-3 PUFAs, as docosahexaenoic acid (DHA) and eicosapentaeinoic acid (EPA), and the omega-6 PUFAs, as arachidonic acid, are available at sites of acute inflammation where they are converted into bioactive SPMs. These SPMs are distinguished in different distinct families, including the omega-6 PUFA-derived lipoxins, and the omega-3 PUFA-derived D-series resolvins, E-series resolvins, protectins, and maresins [8]. The SPMs play a direct key role in the resolution of inflammation, including inhibition of neutrophil migration, enhancement of macrophage phagocytosis of apoptotic neutrophils, and suppression of proinflammatory cytokines and chemokines, in particular during acute illness [1, 9].

Prognosis and outcomes of critically ill patients are directly related to inflammatory status and with the extent and duration of the inflammatory response based on increases in host SPMs, such as resolvin E1, resolvin D5, and 17-epiresolvin D1, and based on survival [10]. Gene expression for SPMs correlated with outcomes in acutely ill patients, and SPM pattern in human tissues was related to outcome in trauma patients [10, 11]. Orr et al. [11] described in trauma patients the blood leukocyte expression of 18 genes involved in the synthesis, signaling, and metabolism of specialized proresolving lipid mediators and proinflammatory lipid mediators. An association was documented between clinical outcomes and gene expression of lipid mediators' pathway, documenting that trauma patients with uncomplicated hospitalization had higher gene expression of resolvin pathway (and lower gene expression of the ratios leukotriene/resolvin pathways), suggesting a potential protective and therapeutic role for SPMs during posttraumatic multiple organ failure [11]. Since EPA and DHA derived from fish oil have shown several health benefits, including improvements in inflammatory conditions and during chronic diseases, as well as a reduction in cardiovascular disease morbidity and mortality and positive neurological effects, scientific interest has been developed in determining whether plant-derived omega-3 PUFA precursors may also present those benefits [5]. Several studies have indicated the potential of plant-derived omega-3 PUFAs to resolve inflammation and to protect against inflammatory diseases, showing that an increased consumption of alpha-linolenic acid (mainly found in flaxseeds, flaxseed oil, and canola oil, also known as rapeseed oil) or stearidonic acid (mainly found in borage and borage seeds and in Corn Gromwell) tends to increase the proportion of EPA and DHA in membranes of inflammatory cells, including neutrophils, monocytes, and lymphocytes [5, 12–15]. It has been documented that parenteral nutrition based on soybean oil, which has also a high content of omega-6 PUFAs and has been largely used over the last decades, may adversely affect the inflammatory response and promote immunosuppressive effects in critical illness [6]. For this reason, alternative lipid emulsion with lower soybean oil content has been used, showing important improvements in clinical outcomes, such as ICU length of stay and mortality [6].

This review focuses on the most recent data on the role of omega-3 PUFAs as pharma nutrition agents in critical illness, and we specifically highlighted their role as anti-inflammatory and proresolving agents.

## 2. Genetic Signature and Inflammation in Critical Illness

Lipid mediators and SPM expression, their biosynthetic isomers, and their biosynthetic pathway reflect the patient's specific genetic background [9]. In particular, Colas et al. recently identified the SPM pathways, including resolvins, protectins, and maresins, in lymphoid tissues, blood, and tissues that were proportional with their regeneration functions and protective and proresolving effects [9]. Endogenous lipid specialized mediators and their pathway of action, involved in regulating/resolving inflammation, are of wide interest, and thus, their genetic signature profiles may provide a functional tool for characterizing health and disease states, as well as in monitoring the impact of treatments [9].

Trauma patients showing complications and worse clinical outcomes have higher expression ratios between leukotriene pathway genes and resolvin pathway genes [11], and in these patients, PUFAs may determine proresolving effects through the modulation of the expression of the genes regulating proinflammatory cytokines [16]. Severe traumatic injury itself may contribute to the dysregulation of lipid mediator pathway gene expression [11]. Clària et al. [17] documented that subcutaneous adipose tissue in patients with peripheral vascular disease had deficient levels of SPMs with potent protective actions in vascular inflammation, indicating phenotypic differences in the capacity and levels of SPMs between adipose tissue from patients with end-stage vascular disease and healthy control subjects.

High omega-3 PUFA circulating concentrations, or the shift in circulating omega-6/omega-3 ratios, might modulate the expression of genes known to be critical during inflammatory processes [16], although stronger clinical evidences are warranted. Experimental evidences have shown that EPA or DHA decrease the expression of genes for interleukin-1-beta and tumor necrosis factor-alpha and their mRNA levels [18–20]. In addition, clinical studies have documented that plant-derived omega-3 PUFAs and combination of long-chain omega-3 PUFAs and a short-chain omega-6 PUFAs were effective in critically ill patients suffering from sepsis, reducing the amount of ventilation time, the number of ICU hospitalization days, and increasing the overall survival [21, 22]. Altering the circulating levels of omega-6 and omega-3 PUFAs may influence the inflammatory responses in part by the capacity of the fatty acids, and specifically their metabolites, to regulate the expression of the early signal transduction genes and to downregulate, at a transcriptional level, the expression of proinflammatory genes involved in inflammatory responses essentially cytokines, chemokines, and NF*κ*B pathway [16, 23]. NF*κ*B is a key transcription factor involved in the upregulation of cyclooxygenase gene, adhesion molecules, and inflammatory cytokines. The study by Allam-Ndoul et al. showed a dose effect of omega-3 PUFAs in inhibiting the gene expression of selected inflammatory cytokine and of genes involved in the NF*κ*B pathway [23].

Additional studies in critical setting support the potential protective and therapeutic role for SPMs in reducing complications in posttraumatic conditions. Experimental evidences showed that survival improved significantly after administration of resolvins to septic and burn-injured animals [24]. Similarly, clinical randomized controlled trials documented that intravenous administration of oil, containing DHA and EPA, may decrease mortality and ventilator days in critically ill patients [25, 26], although most recent randomized controlled trials, conducted in critically ill adult patients, did not document an effect in improving overall mortality [27].

It appears important to assess whether pathological conditions, characterized by excessive inflammation, such as critical illness, result from failed resolution mechanisms because of lack or block of specific SPM pathways and whether these mechanisms may be modulated either by EPA or DHA supplementation, or by therapies mimicking SPMs [28].

Patient's profiling of SPM pathway(s) may allow for the identification of metabolites possibly serving as proresolving mediators [9].

## 3. How Inflammation Is Biologically Resolved in ICU?

An old mechanistic vision considered the resolution of inflammation as a passive process at the end characterized by decreased levels of cytokines, prostaglandins, and reactive oxygen species. During sepsis or other major inflammatory stresses, there is a balance within the host organism. The proinflammatory pathways rise to eliminate pathogens and dead tissue, often causing injury to the host. The anti-inflammatory responses, such as the systemic inflammatory response syndrome (SIRS) and the compensatory anti-inflammatory response syndrome (CARS) (coexistence of both is referred to as mixed antagonist response syndrome (MARS)), seem to limit the damage not interfering with the pathogen elimination. Nevertheless, CARS response may be dangerous when its effects are poorly timed, causing leukopenia, susceptibility to infection, and failure to clear infection [29].

Conversely, during the past few years, the resolution of inflammation has been more clearly identified as an active process where lipid mediators specifically participate in the resolution process by switching their phenotype [30].

**Figure 1:**
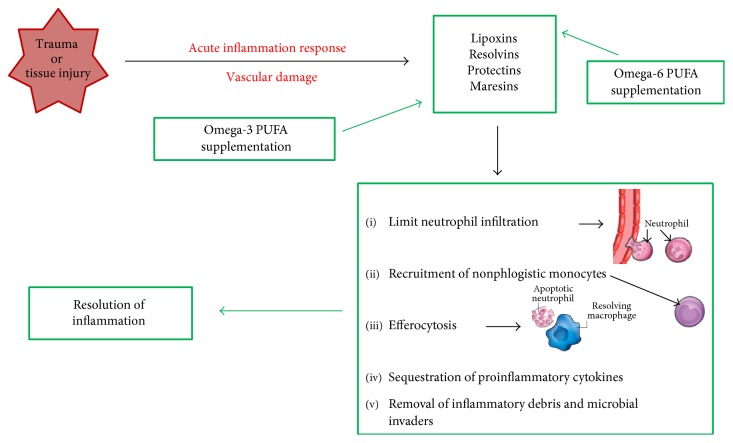
After a trauma or tissue injury, there is a vascular damage inducing the acute inflammatory response. The specialized lipid mediators (SPMs), derived from omega-6 and omega-3 fatty acid storage, act as proresolving mediators. The SPM class which initiates to resolve inflammation is represented by lipoxins that are able to limit neutrophil infiltration. Lipoxins and resolvins stimulate the recruitment of nonphlogistic monocytes. Resolvins and protectins stimulate the resolving macrophages to clear apoptotic neutrophils in the efferocytosis process. Signs of resolution include sequestration of proinflammatory cytokines and removal of inflammatory debris and microbial invaders. Maresins stimulate reepithelialization, wound healing, and tissue regeneration. Omega-3 fatty acid supplementation may enhance proresolving inflammatory responses via their capacity to regulate the expression of proinflammatory cytokines through the production of SPMs. PUFAs, polyunsaturated fatty acids.

Lipid mediators have a crucial role in the vascular damage and leukocyte recruitment, from initiation to resolution of inflammation [28]. Eicosanoids derived from omega-6 PUFAs are potent proinflammatory mediators, except for lipoxins, which are able to perform local proresolving actions in association with lipid mediators obtained from omega-3 PUFAs [9]. Lipoxins, resolvins, protectins, and maresins are produced during response to inflammation, as signaling molecules, and play a role in resolving inflammatory exudates (Figure 1). As part of the neutrophil-monocyte sequence, the lipoxin signals promote the blocking of the acute inflammatory response. Lipoxins and resolvins stimulate the recruitment of nonphlogistic monocytes. So, the resolving macrophages clear the apoptotic neutrophils. In this light, SPMs regulate the actions of the classic proinflammatory initiators prostaglandins and leukotrienes [9], reducing the duration of inflammation, and stimulate reepithelialization, wound healing, and tissue regeneration as signs of resolution [28].

Epidemiological studies and several randomized control trials demonstrate a positive relationship between consumption of omega-3 PUFAs (specifically EPA and DHA) and improvements of different inflammatory conditions [5]. Serhan et al. [7] demonstrated that increasing cellular uptake of omega-3 PUFAs causes an enhancement in the production of resolvins and protectins, which are crucial in resolving inflammatory responses (Figure 1).

An increasing interest is nowadays present in the study of the metabolism, functional effects, and health benefits of omega-3 PUFAs derived from plants [5].

In particular, DHA and EPA act through their enzymatic conversion to the potent lipid-derived mediators [31, 32]. DHA is enzymatically converted to the D-series resolvins through transcellular biosynthesis [1] and intermediates to the protectin family through 15-lipoxygenase action via an epoxide-containing intermediate [33]. The third major family of DHA-derived SPMs is represented by maresins, with a potent proresolving and tissue regenerative action, synthetized by macrophage involving an epoxide-maresin intermediate [28]. EPA can be enzymatically converted into the E-series resolvin family of SPMs through transcellular mechanisms [1].

Several reports in experimental models demonstrated important roles for SPMs in promoting a return to homeostasis after infection or injury leading to improved outcomes and survival [1].

SPMs have shown positive effects in decreasing pain and risk of sepsis, increasing epithelialization and wound healing, inducing tissue regeneration, potentiating the effects of antibiotics, and enhancing adaptive immunity [8]. Nevertheless, further and strong evidences are needed to clarify the effects and potential benefits of the use of SPMs in critical care, including a comparison with the effects obtained by the administration of oil-derived omega-3 PUFAs.

## 4. Clinical Aspect of the Anti-Inflammatory or Proresolving Effect of PUFAs

Since long time, several authors have discussed the anti-inflammatory effect of omega-3 PUFAs, acting through different mechanisms, including pathways via the cell membrane (G-protein coupled receptor 120) and intracellular (peroxisome proliferator-activated receptor (PPAR) gamma) receptors that control inflammatory cell signaling and gene expression patterns [34]. Therefore, EPA and DHA were considered as anti-inflammatory agents, whereby they compete with omega-6 arachidonic acid, reducing proinflammatory molecules [28]. In fact, in the last year's research, focus has shifted from inhibiting inflammation to accelerating resolution of inflammation using SPMs [8]. The anti-inflammatory process is not the same as proresolution, which involves the SPMs in activating the nonphlogistic responses and cell resolution programs [28]. The proresolving actions include inhibition of neutrophil tissue infiltration, counter-regulation of chemokines and cytokines, reduction in pain, and stimulation of actions mediated by macrophages, known as efferocytosis and phagocytosis of microbes [35] (Figure 1) (Table 1).

Supplementation of omega-3 PUFAs alters the profile of proinflammatory cytokine expression and production and provides significant modifications in inflammatory response aimed at resolving this process [16]. In critical illness, SPMs seem to regulate the excessive posttrauma inflammatory response and to protect organs from collateral damage [28], offering a potential therapeutic option modulating inflammation with minimal side-effects in contrast to currently available anti-inflammatory therapies.

The use of parenteral omega-3 PUFA-based lipid emulsions was considered safe and effective in ICU patients and in the postoperative period in terms of reduction in the infection rate lengths of ICU and in-hospital overall stay, while no significant difference in the mortality rate was documented between patients receiving omega-3 PUFA-enriched parenteral emulsions and those receiving standard lipid emulsions [36]. The latest clinical meta-analysis results in ICU patients documented a reduction of infections and a reduction in hospital length of stay in cardiac surgery spatients [37].

More recently, Grau-Carmona et al. investigated the effects of omega-3 PUFAs on the prevalence of nosocomial infections and clinical outcomes in medical and surgical critically ill patients [38]. The number of patients with nosocomial infections was significantly reduced in the group receiving the omega-3 PUFA supplementation and the predicted time free of infection was prolonged, showing that the administration of fatty acids was safe and well tolerated and that it reduced the risk of nosocomial infections and increased the predicted time free of infections in critically ill medical and surgical patients (Table 1). However, no statistically significant differences were observed on the length of ICU and hospital stay, days of ventilation, and mortality [38].

In addition, Pradelli et al. evaluated the cost-effectiveness of the addition of omega-3 PUFAs to standard parenteral nutrition regimens in four European countries (from the healthcare provider perspective) [39]. The authors concluded that, according to their results, the supplementation of parenteral nutrition regimens with omega-3 PUFAs would be cost-effective in Italian, French, German, and United Kingdom hospitals [39].

In conclusion, resolution of inflammation is an active process, mainly driven by the synthesis of PUFA-derived SPMs. It is becoming increasingly clear that omega-3 PUFAs are both anti-inflammatory and proresolving nutrients. In fact, EPA and DHA not only act as anti-inflammatory agents, according to the classical view (i.e., by competing for the synthesis of proinflammatory, omega-6-derived mediators) but also actively promote the resolution of inflammation through the synthesis of SPMs. Administration of fatty acids affects SPM levels in plasma, the immune function, and it may be associated with better outcome and reduce health costs in surgical and acutely ill patients (Table 1). However, controversy still exists on the indications for the use of specific lipid emulsion(s) in ICU patients [6, 36, 38]. In the near future, the possibility to assess circulating SPM levels before, during, and after omega-3 PUFA supplementation, as well as the administration of SPMs, will possibly allow to assess the efficacy of the treatment and to better clarify the mechanisms through which omega-3 PUFAs and PUFA-derived mediators may confer clinical benefit in critically ill patients.

## Figures and Tables

**Table 1:** Key points: proresolving effects of omega-3 fatty acids in critical illness.

Omega-3 fatty acids, principally present in dietary fish oils, are derived also from plants and are able to reduce or limit inflammation during disease, including acute and critical illness [4–6].
(ii) The biological effects of omega-3 fatty acids are mediated by the production of specialized proresolvin mediators (SPMs) [8, 9].
(iii) Gene expression of SPMs in human tissues correlates with outcomes in critically ill patients [10, 11, 17].
(iv) Lipoxins, resolvins, protectins, and maresins are SPMs produced in response to inflammation, able to accelerate resolution of inflammation rather than inhibiting inflammation [4, 7–9].
(v) Key biologic actions of SPMs are to limit neutrophil infiltration, promote efferocytosis of apoptotic cells, enhance microbial clearance, counter-regulate cytokines and chemokines, and downregulate prostanoids [10, 30, 33, 34].
(vi) Administration of omega-3 fatty acids in surgical and acutely ill patients may be associated with better outcome and reduced health costs.
